# Supplementary material for: Proliferation, Migration and Invasion of Breast Cancer Cell Lines Are Inhibited by 1,5-Disubstituted Tetrazol-1,2,3-triazole Hybrids through Interaction with p53
Source: Molecules. 2023 Nov 15;28(22):7600. doi: 10.3390/molecules28227600 (PMC10673467; doi:10.3390/molecules28227600)
Supplement: Supplementary file 1 [file molecules-28-07600-s001.zip › molecules-2646020-supplementary.pdf]

Supporting Information

A one-pot six component reaction for the synthesis of 1,5-disubstituted tetrazol-1,2,3-triazoles hybrid and their cytotoxic activity against the MCF-7 cell line

Cesia M. Aguilar-Morales [1], Jorge G. Araujo-Huitrado[2], Yamilé López-Hernandez[3], Claudia Contreras-Celedón[1], Alejandro Islas-Jácome[4], Angelica Judith Granados-López[2], Cesar Rogelio Solorio-Alvarado[5], Jesús AdriánLópez[2], Luis Chacón-García[1]*, and Carlos J. Cortés-García [1,]*.

[1] Laboratorio de Diseño Molecular, Instituto de Investigaciones Químico-Biológicas, Universidad Michoacana de San Nicolás de Hidalgo. Ciudad Universitaria, C.P. 58033, Morelia, Michoacán, México; cesia_aguilar@hotmail.com (C.M. A.-M.); claudia.contreras@umich.mx (C. C.-C.)

[2] Laboratorio de microRNAs y Cáncer, Universidad Autónoma de Zacatecas, Av. Preparatoria S/N, Agronómica, Campus II, C.P. 98066, Zacatecas, Zacatecas, México; aahj011871@uaz.edu.mx (J. G. A.-H.); agranadosjudith@gmail.com (A. J. G.-L.); jalopez@uaz.edu.mx (J. A.-L.)

[3] Laboratorio de Metabolómica y Proteómica, Cátedra CONACYT, Universidad Autónoma de Zacatecas, Av. Preparatoria S/N, Agronómica, Campus II, C.P. 98066, Zacatecas, Zacatecas, México aahj011871@uaz.edu.mx (ylopezher@conacyt.mx (Y. L.-H.).

[4] Departamento de Química, Universidad Autónoma Metropolitana-Iztapalapa, San Rafael Atlixco 186, Col. Vicentina, C.P. 09340, Iztapalapa, Ciudad de México; aij@xanum.uam.mx (A. I.-J.)

[5] Departamento de Química, Division de Ciencias Naturales y Exactas, Universidad de Guanajuato, Campus Guanajuato, Noria Alta S/N, 36050, Guanajuato, Guanajuato, México; csolorio@ugto.mx (C. R. S.-A.)

TABLE OF CONTENTS

NMR spectra of the products **7a-o**

Note: The enumeration of compounds **13** in the original supporting information was changed to number **7**, which corresponds to this manuscript.

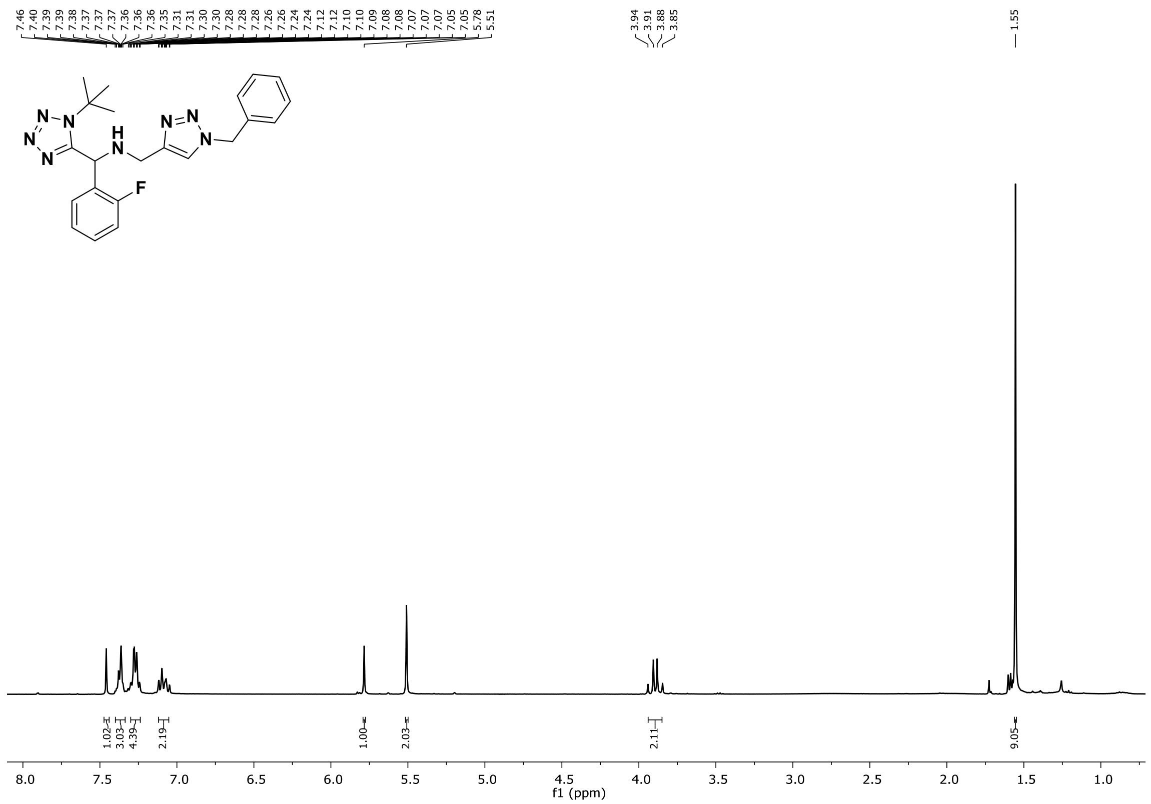

^{1}H NMR spectra of the compound **7a**

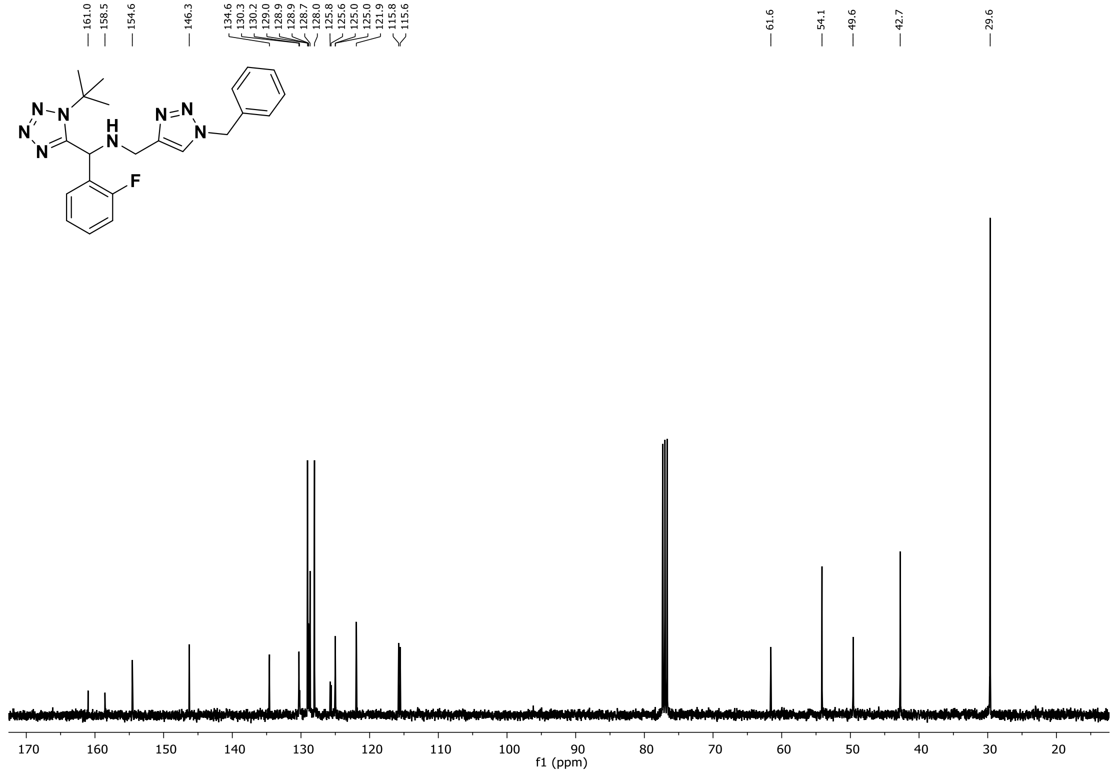

^{13}C NMR spectra of the compound **7a**

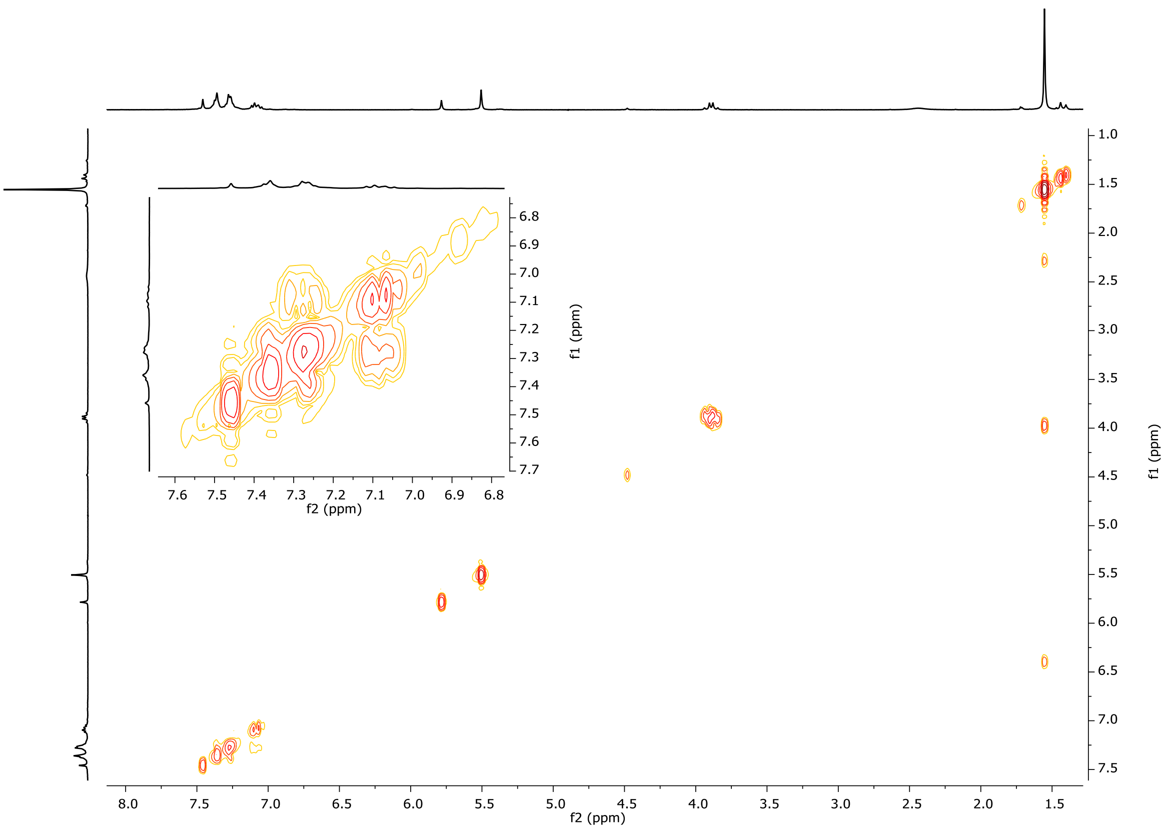

COSY spectra of the compound **7a**.

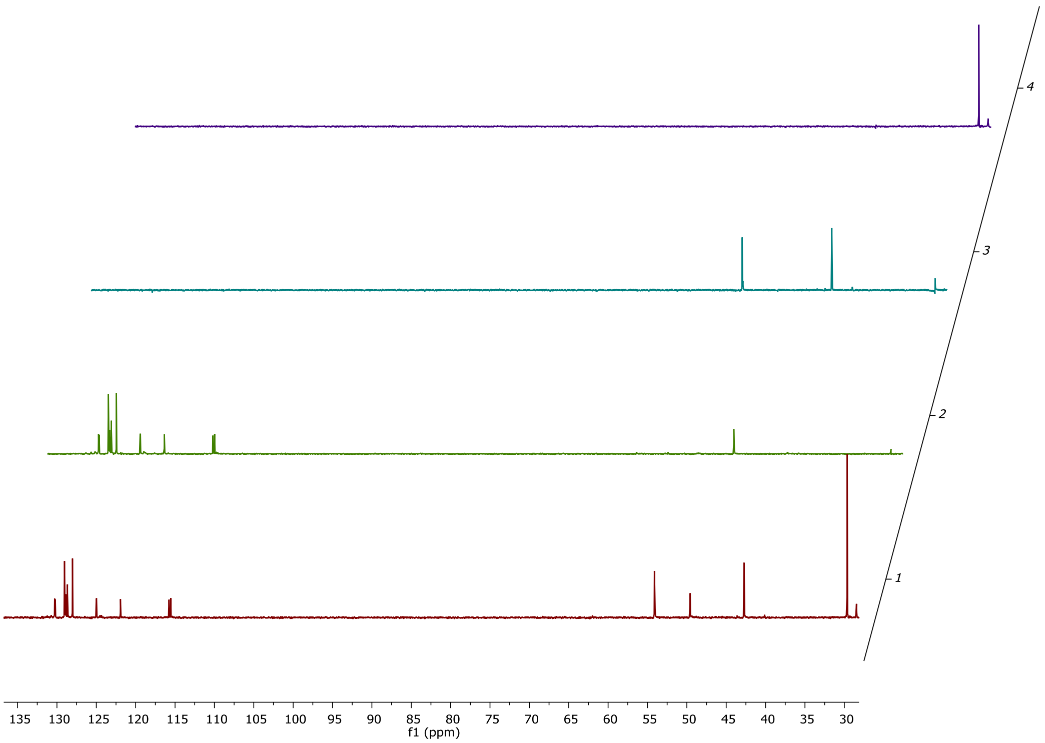

DEPT spectra of the compound **7a**.

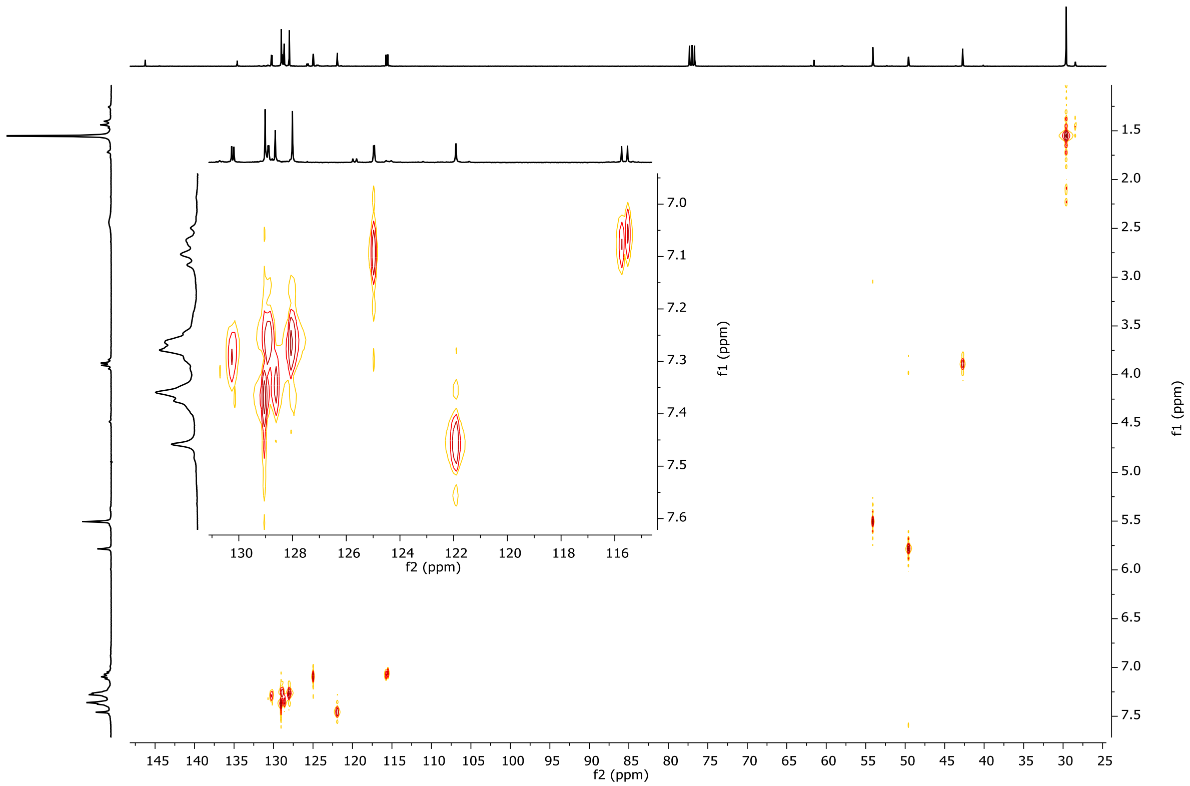

HETCOR spectra of the compound **7a**.

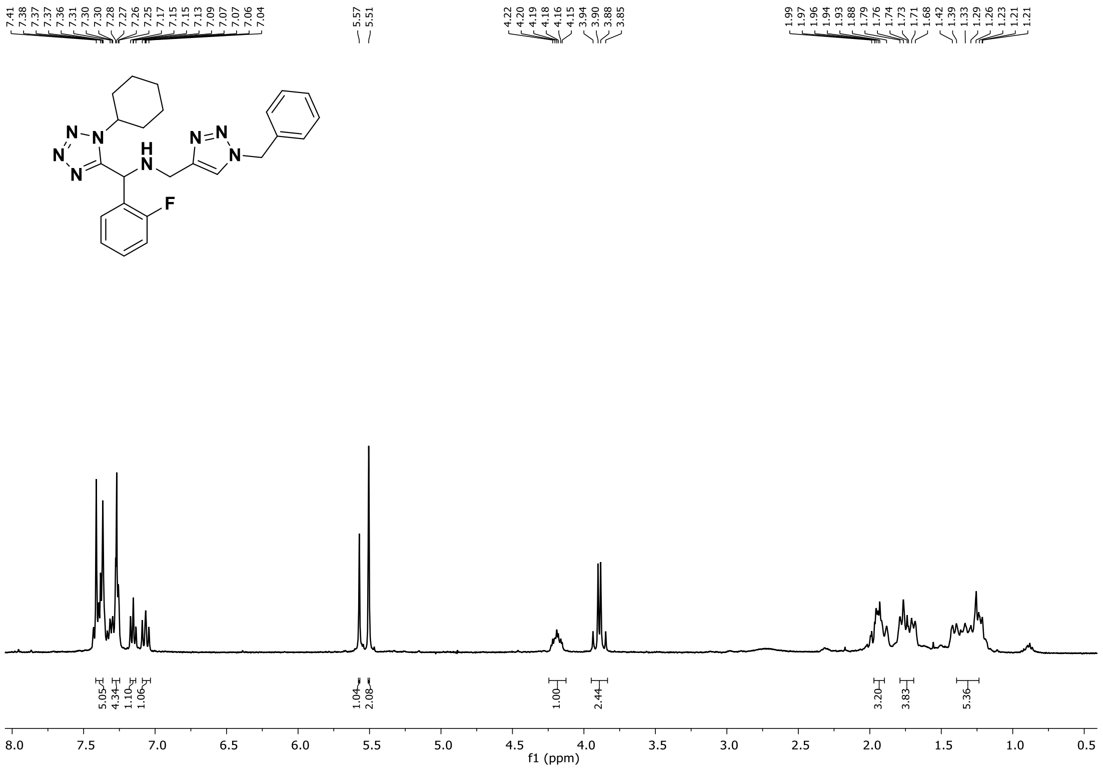

^{1}H NMR spectra of the compound **7b**.

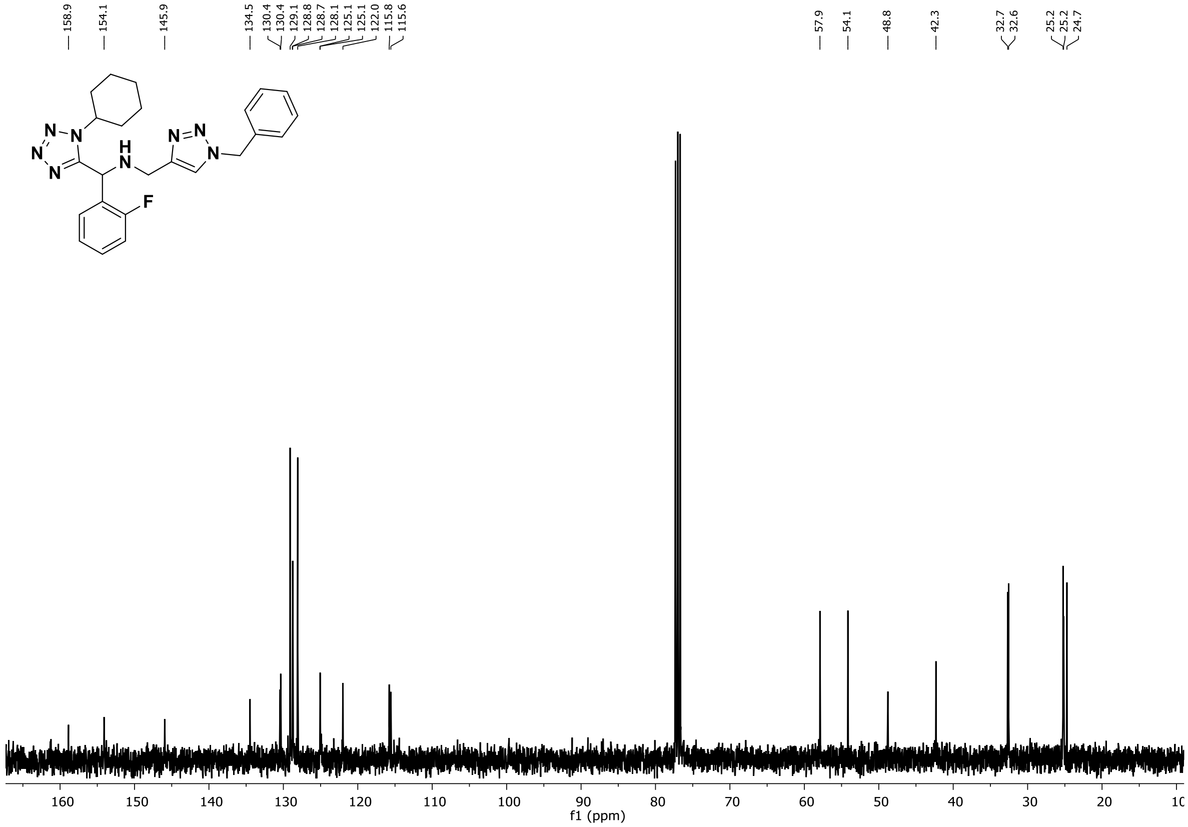

[13] C NMR spectra of the compound **7b**.

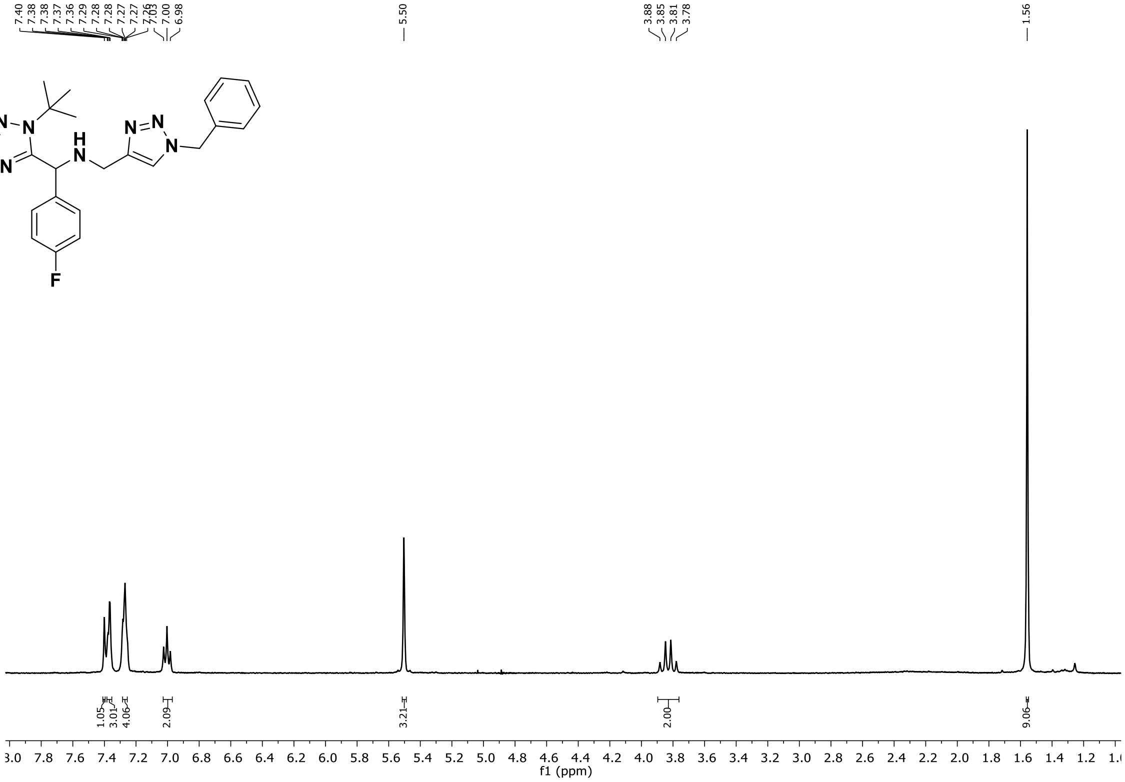

^{1}H NMR spectra of the compound 7**C**.

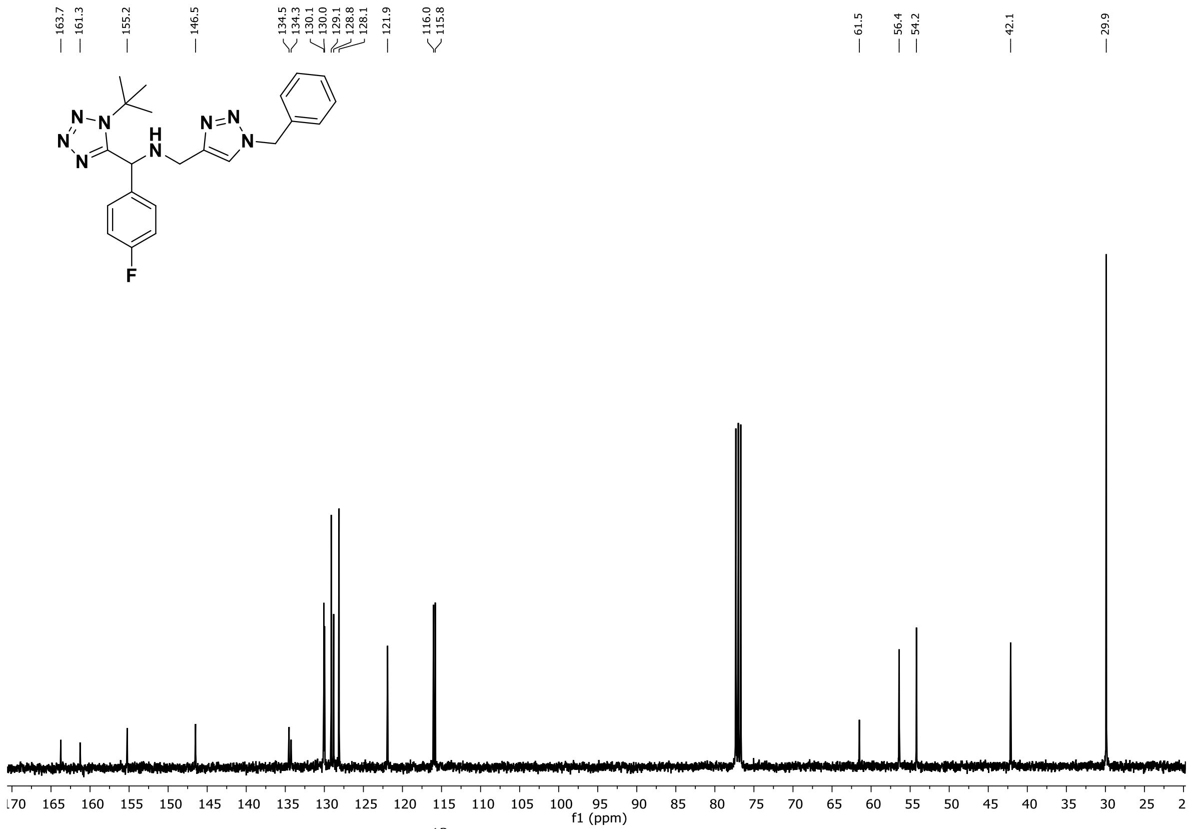

^{13} C NMR spectra of the compound **7C**.

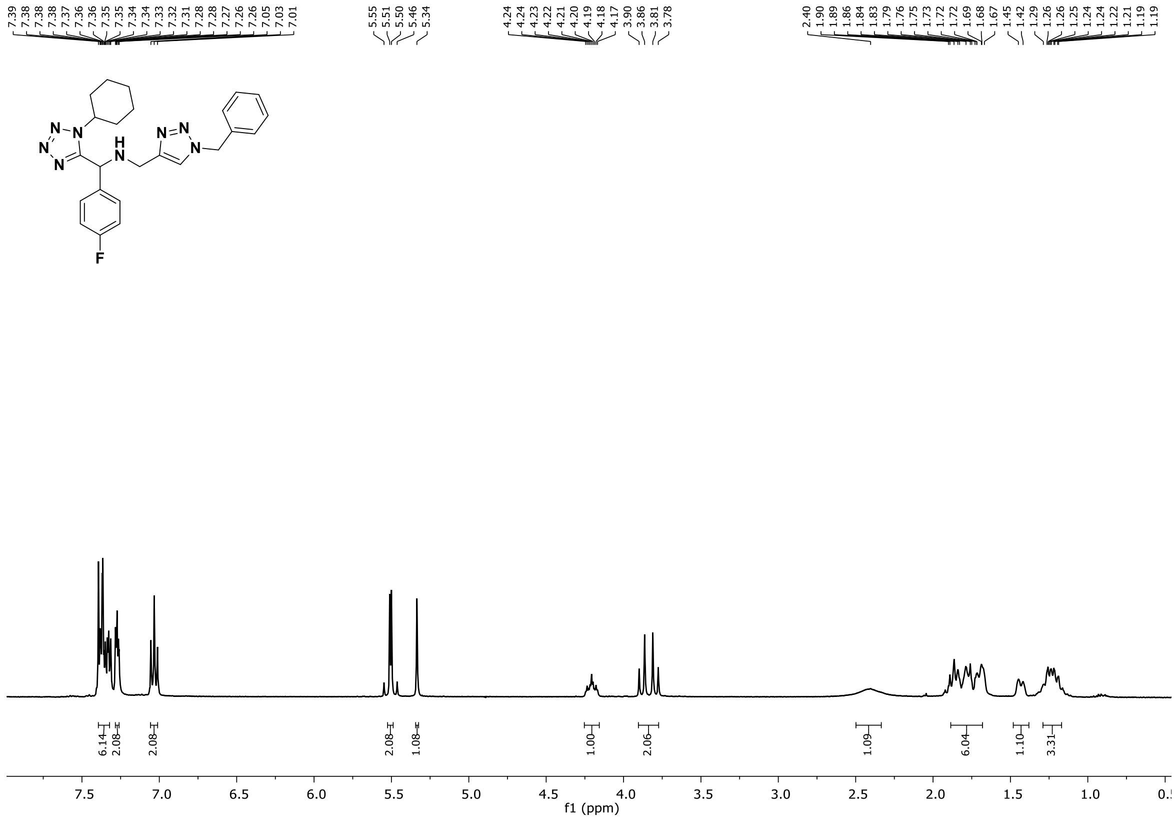

^{1}H NMR spectra of the compound **7d**.

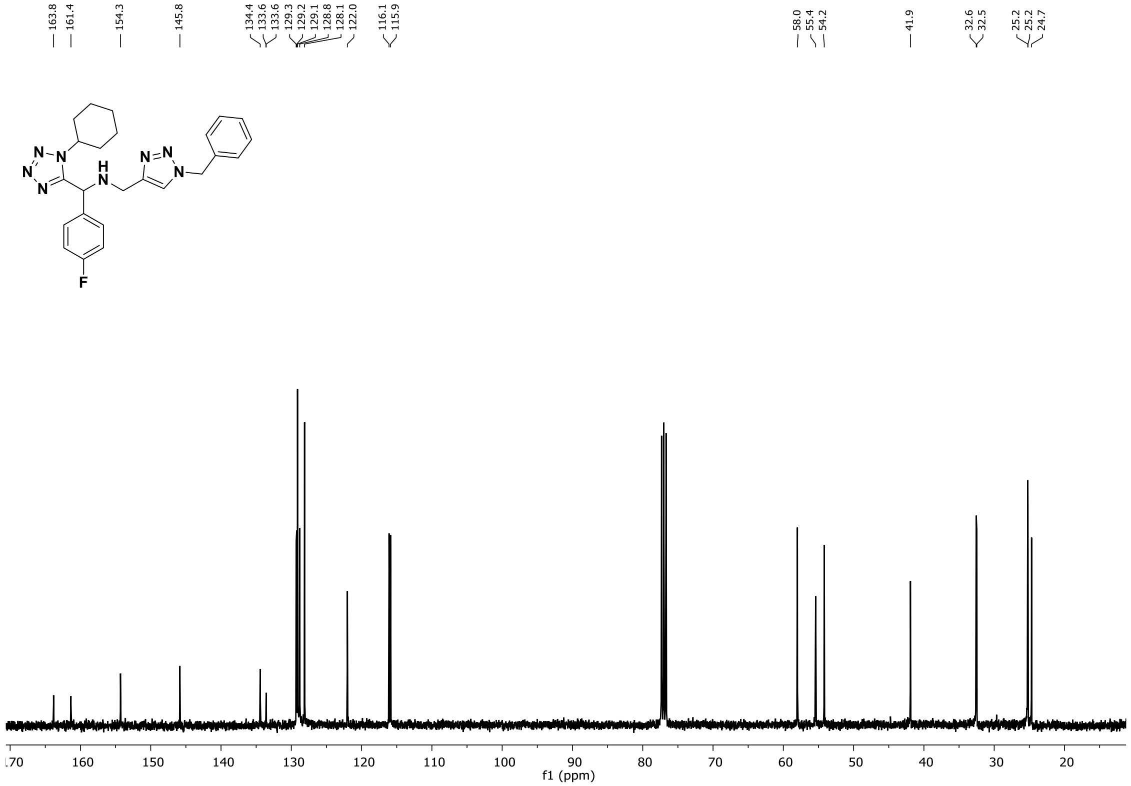

^{13} C NMR spectra of the compound **7d**.

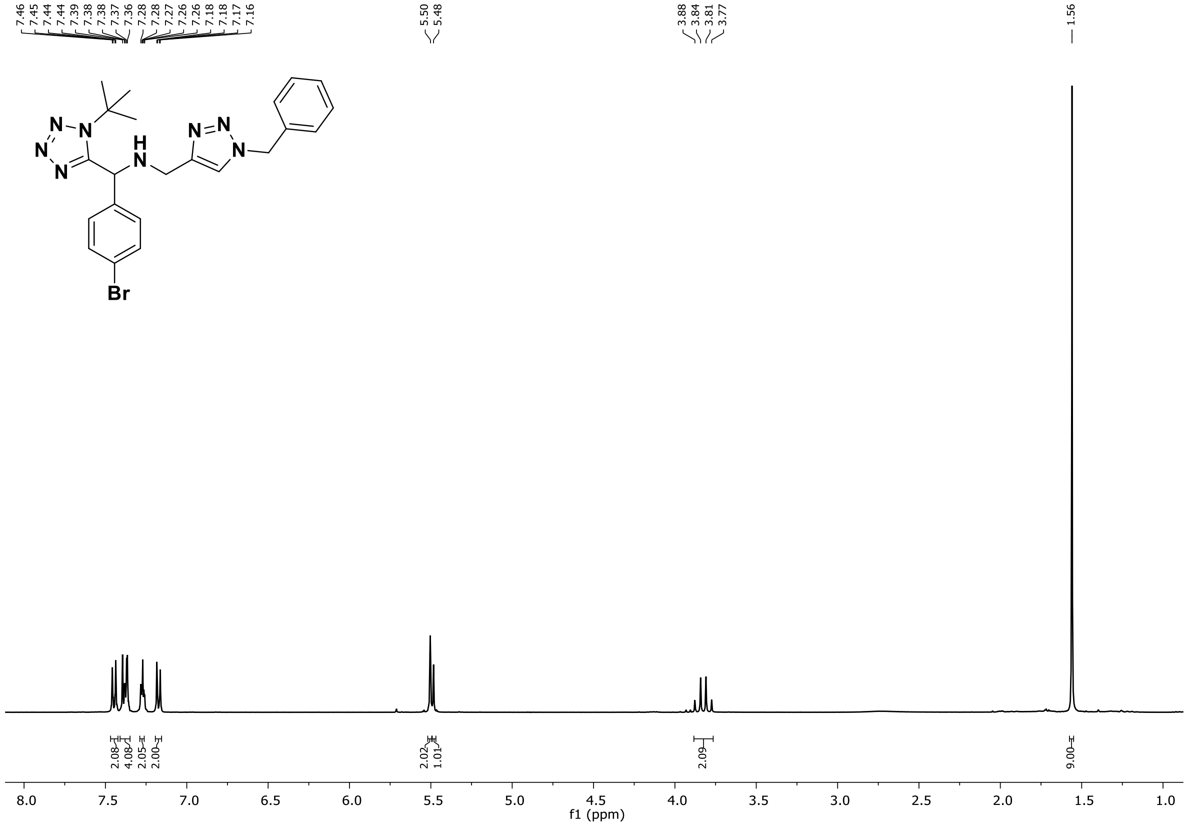

^{1}H NMR spectra of the compound **7e**.

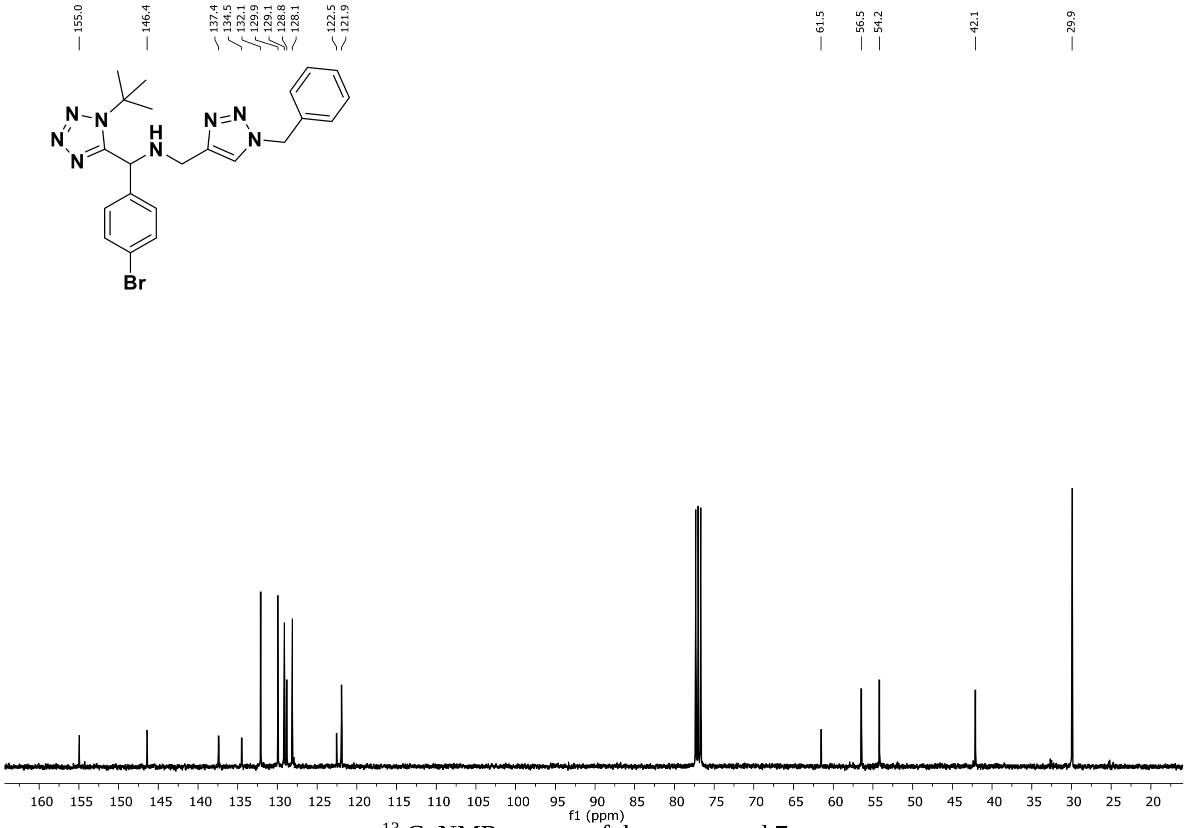

^{13}C NMR spectra of the compound **7e**.

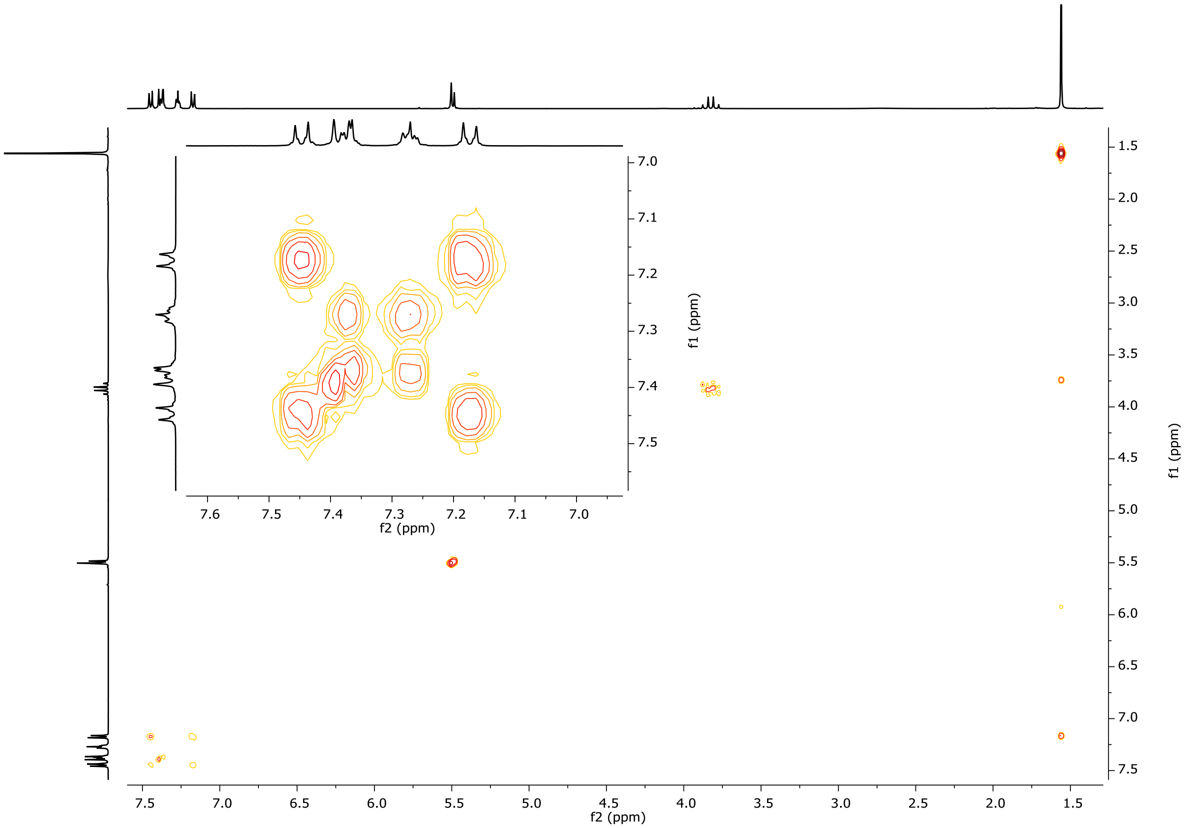

COSY spectra of the compound **7e**.

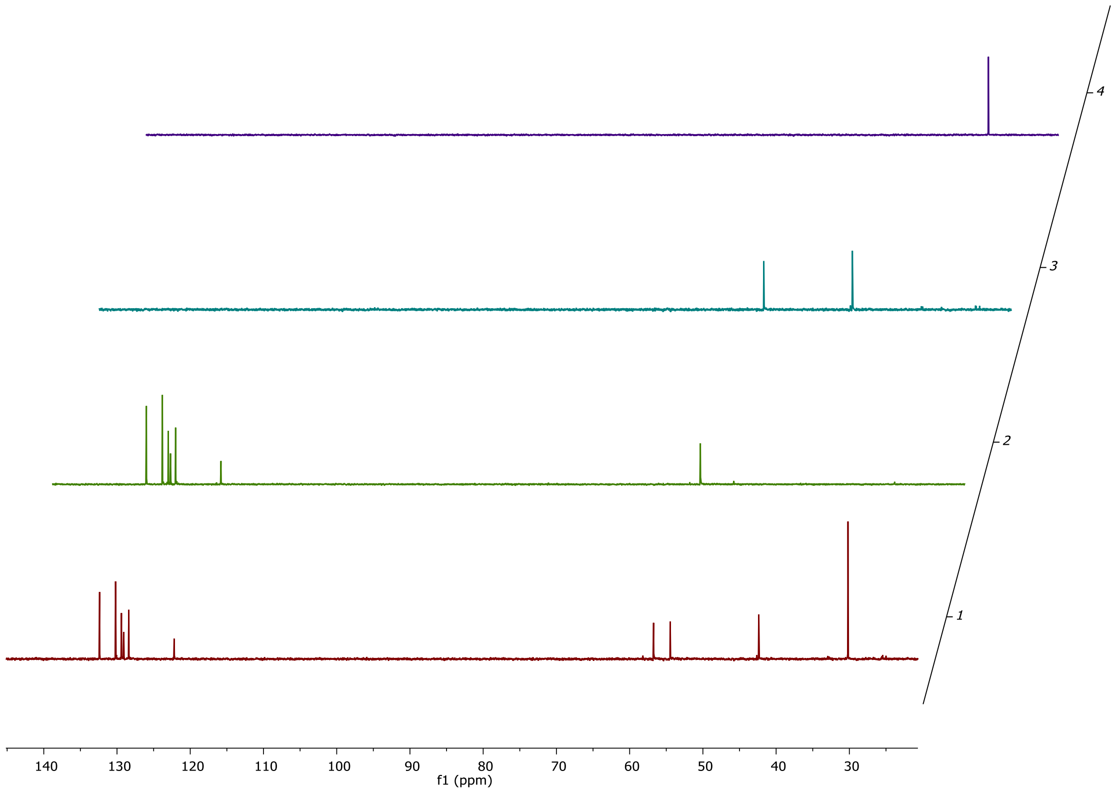

DEPT spectra of the compound **7e**.

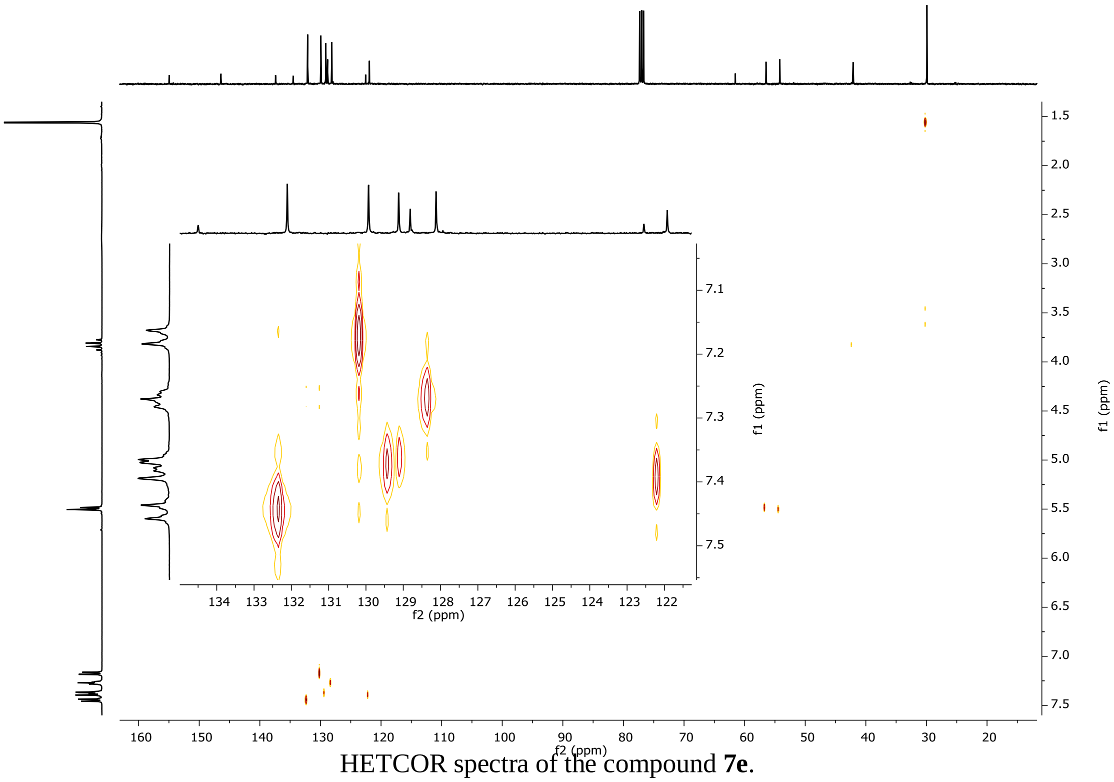

HETCOR spectra of the compound **7e**.

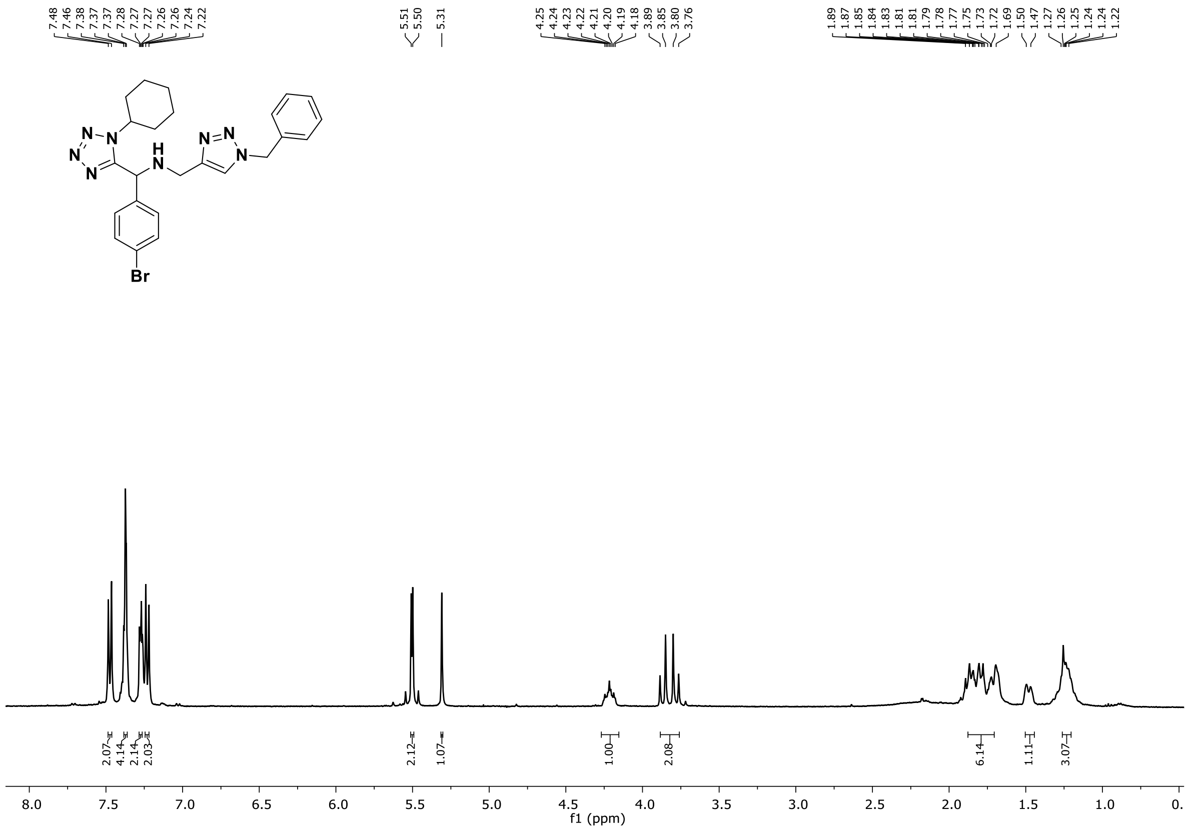

^{1}H NMR spectra of the compound **7f**.

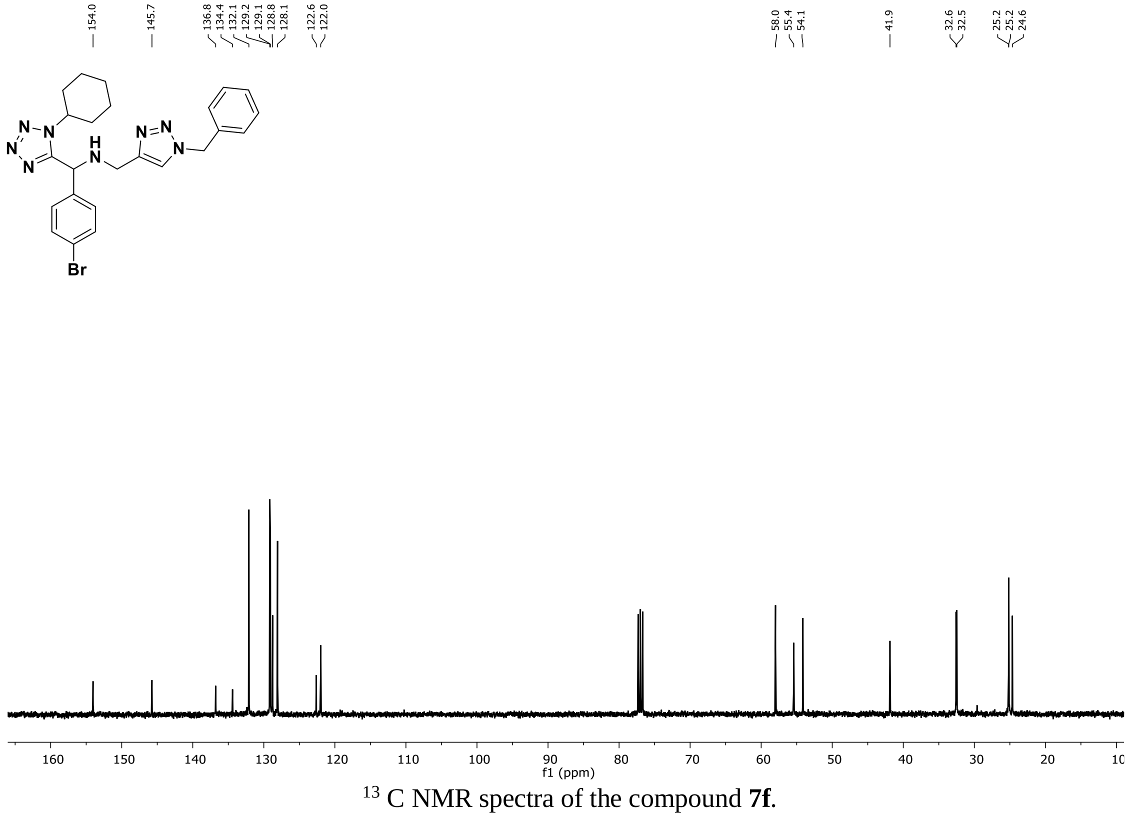

[13] C NMR spectra of the compound **7f**.

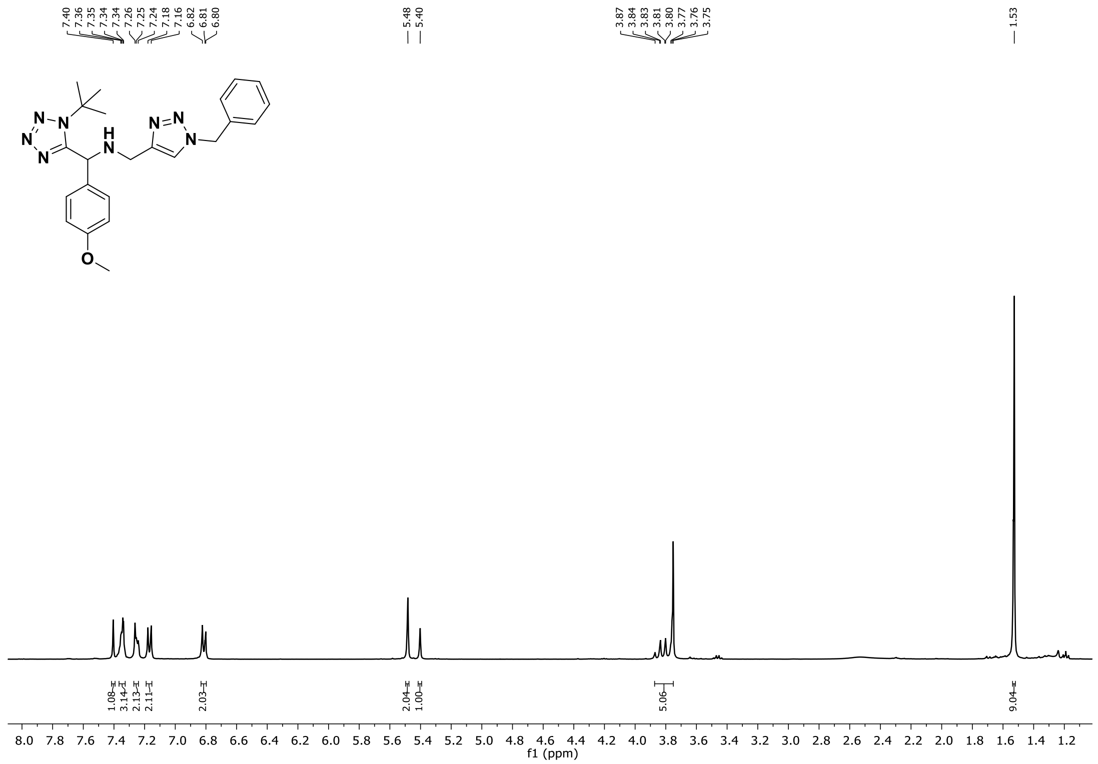

^{1}H NMR spectra of the compound **7g**.

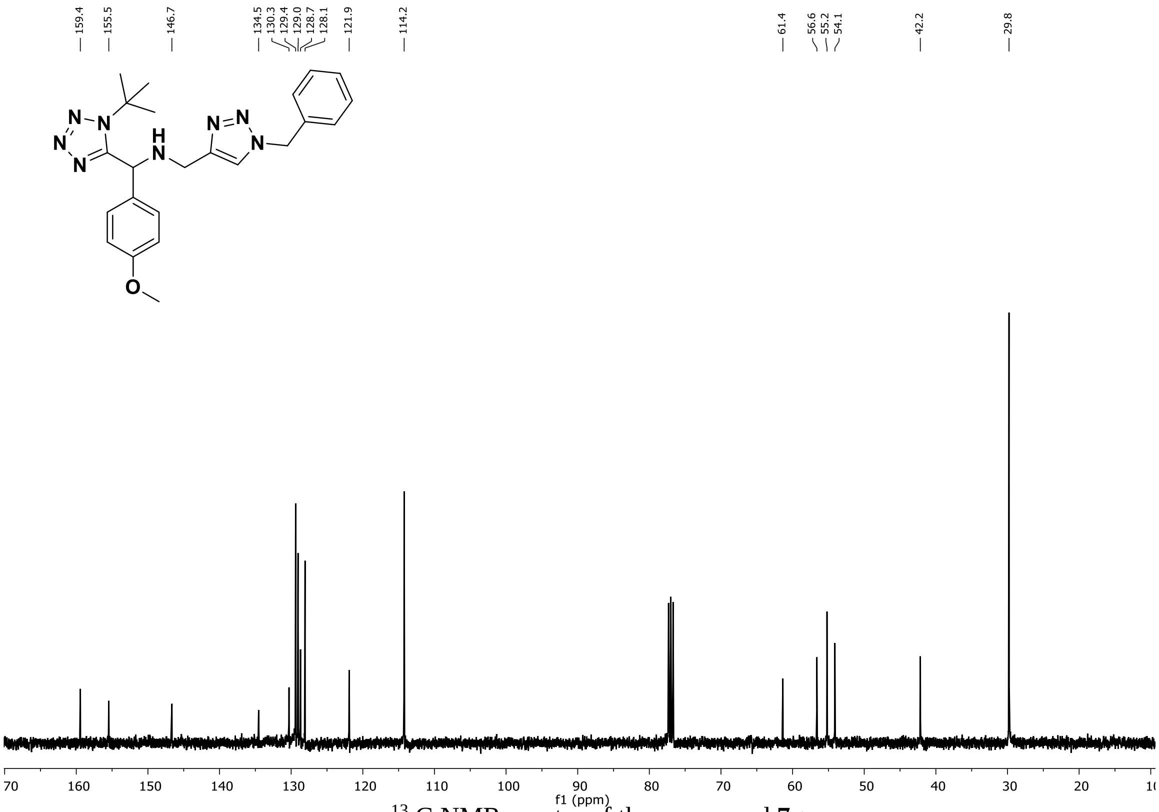

^{13} C NMR spectra of the compound **7g**.

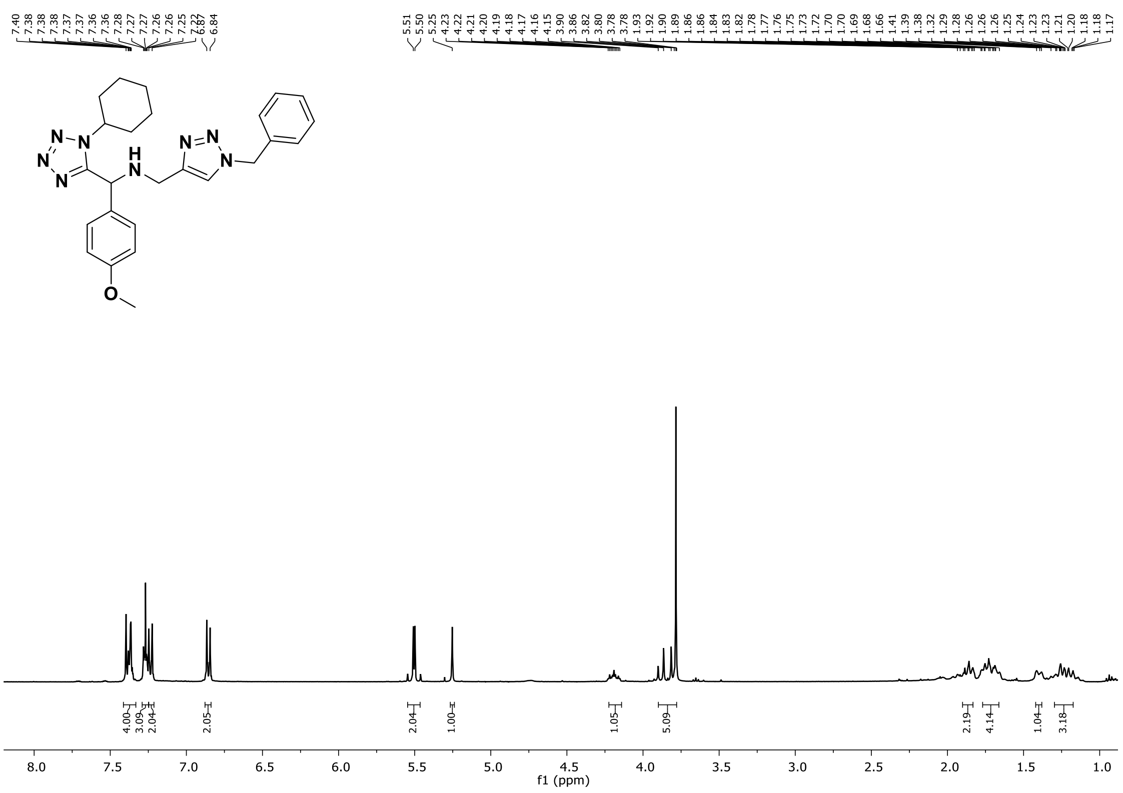

^{1}H NMR spectra of the compound **7h**.

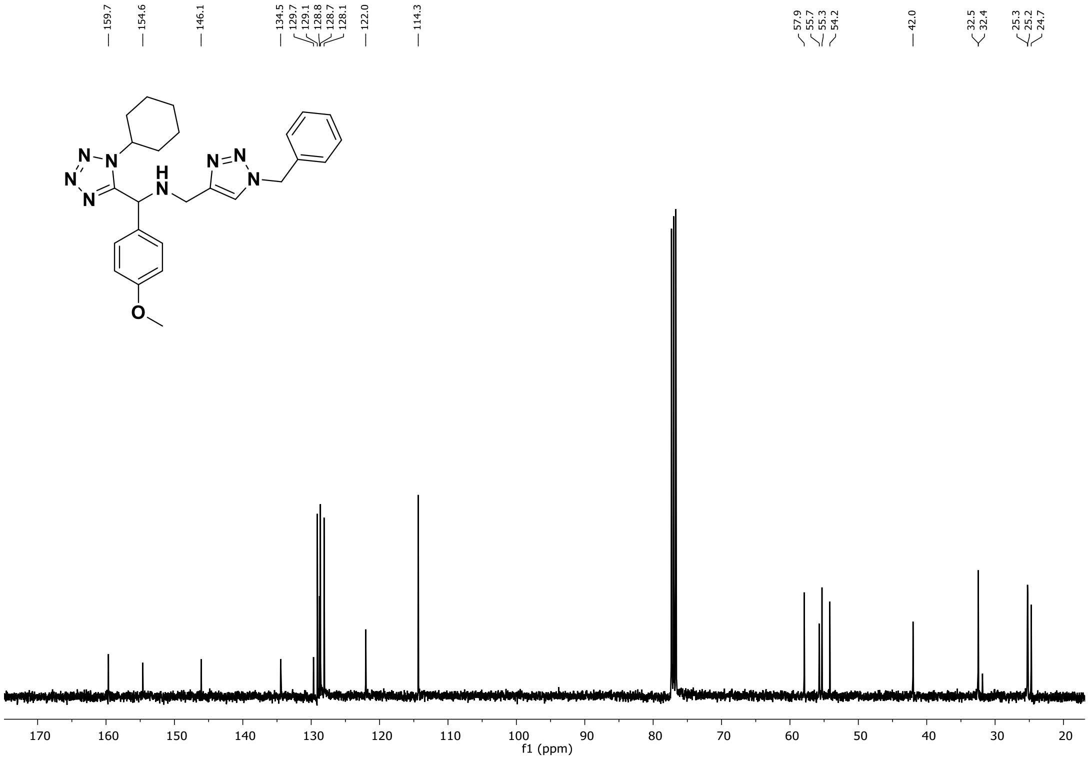

[13] C NMR spectra of the compound **7h**.

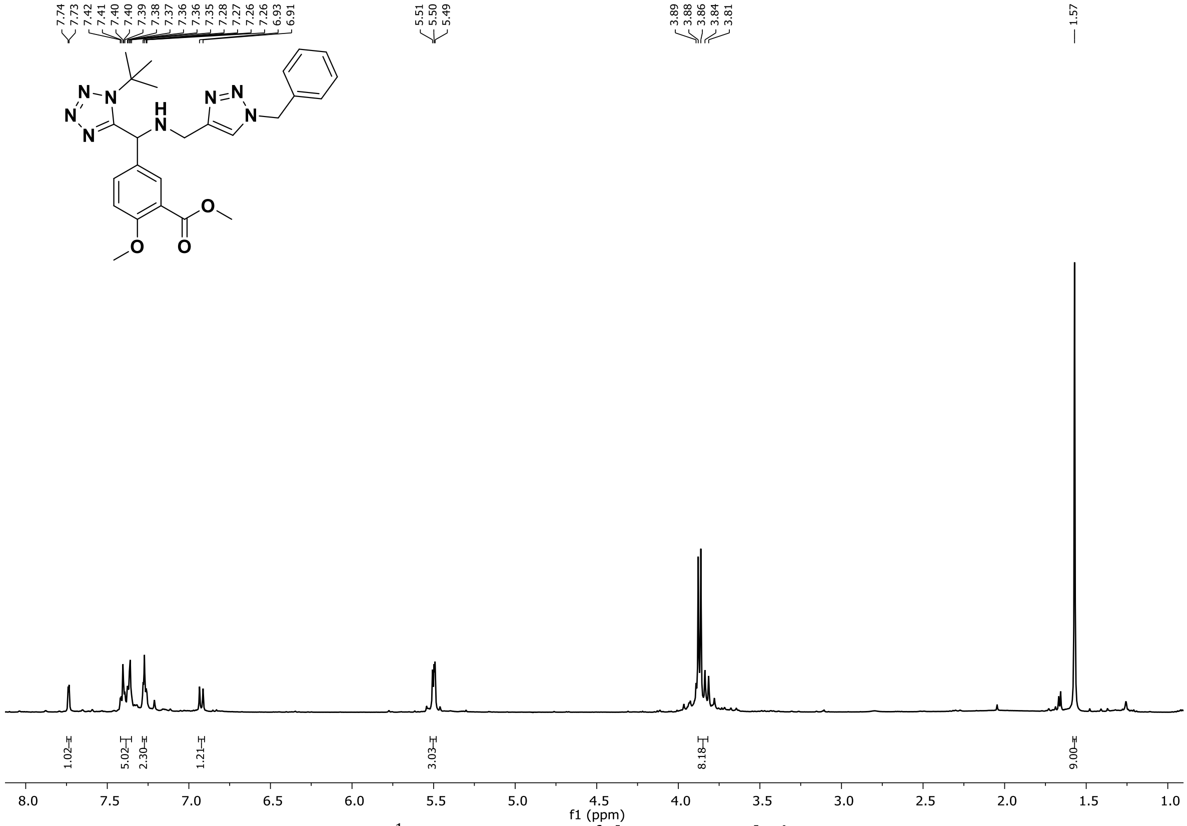

^{1}H NMR spectra of the compound **7i**.

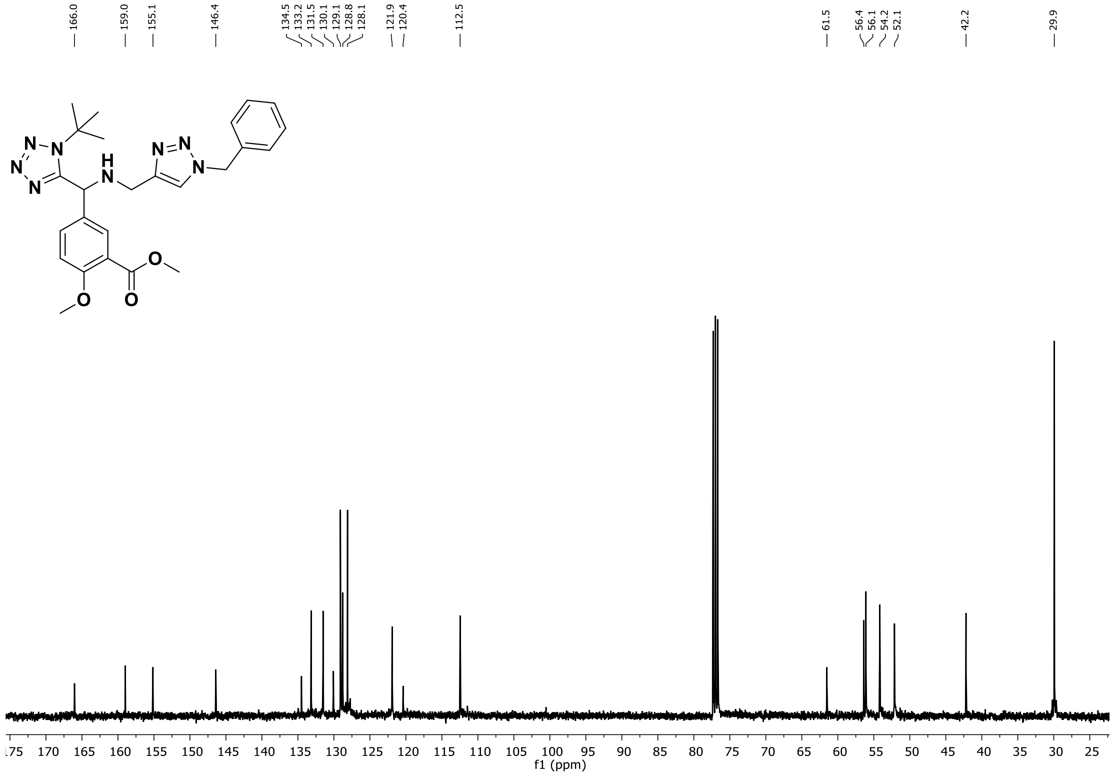

[13] C NMR spectra of the compound **7i**.

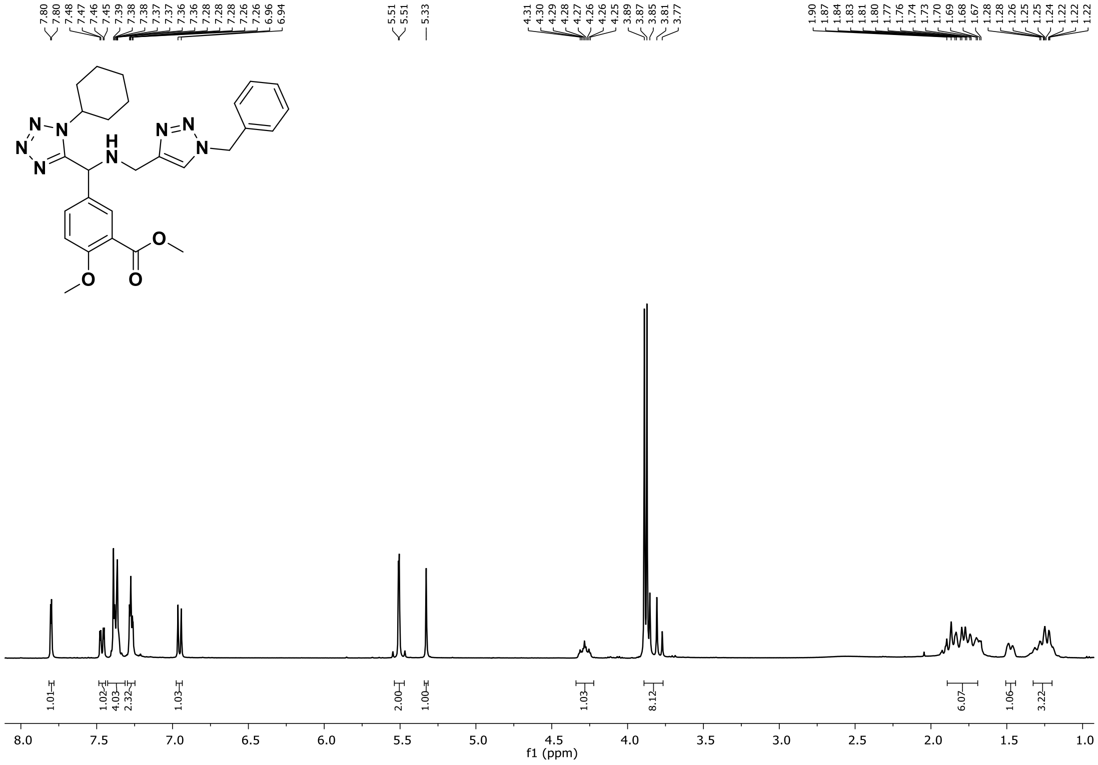

^{1}H NMR spectra of the compound **7j**.

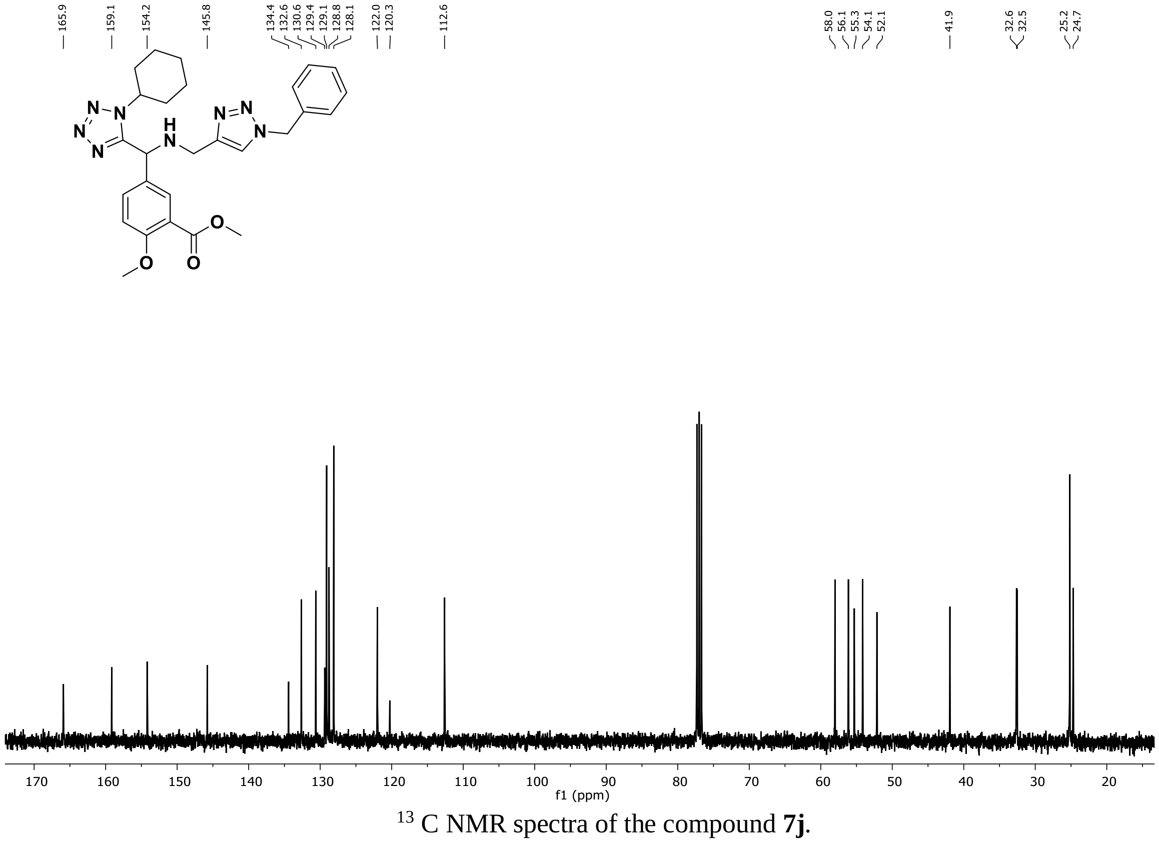

^{13} C NMR spectra of the compound **7j**.

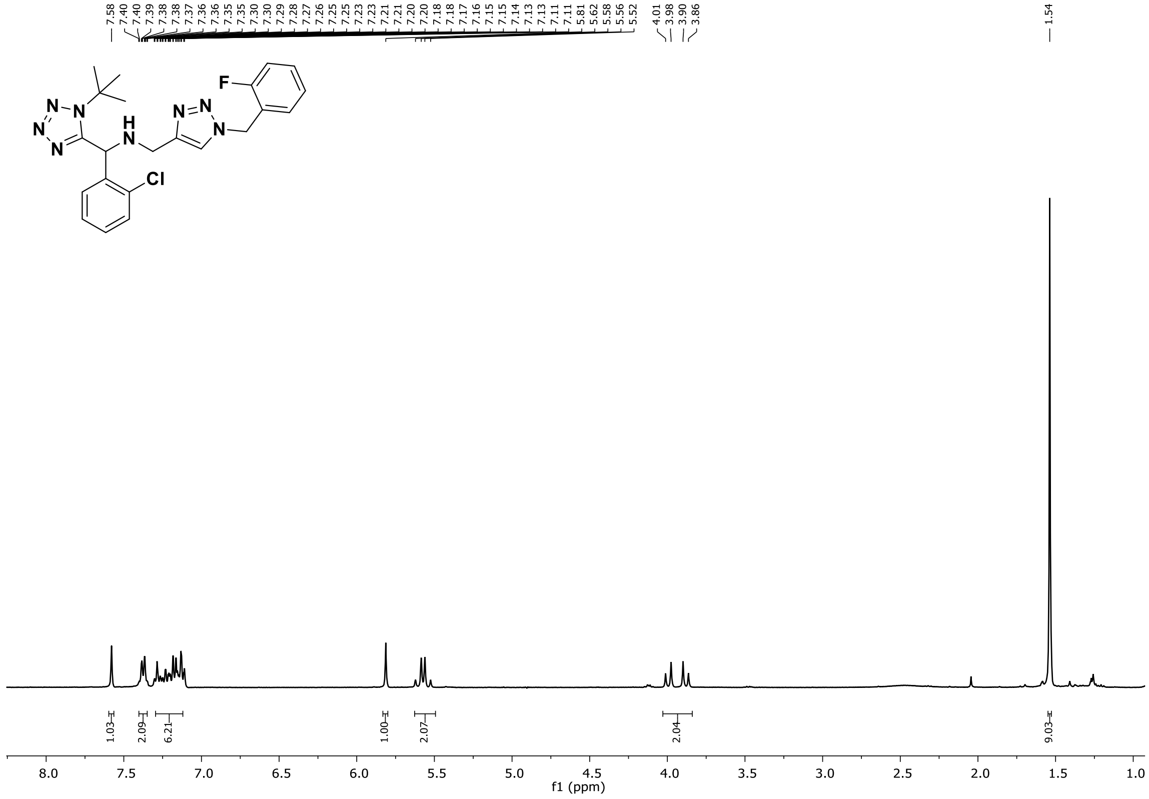

^{1}H NMR spectra of the compound **7k**.

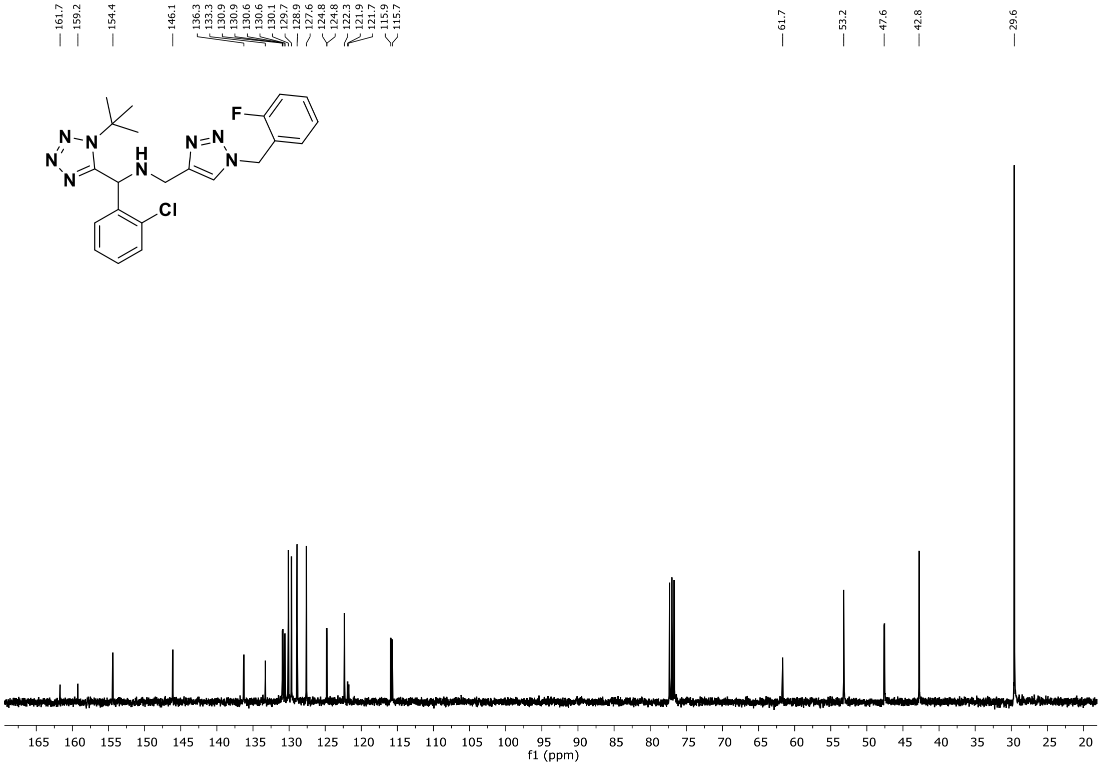

[13] C NMR spectra of the compound **7k**.

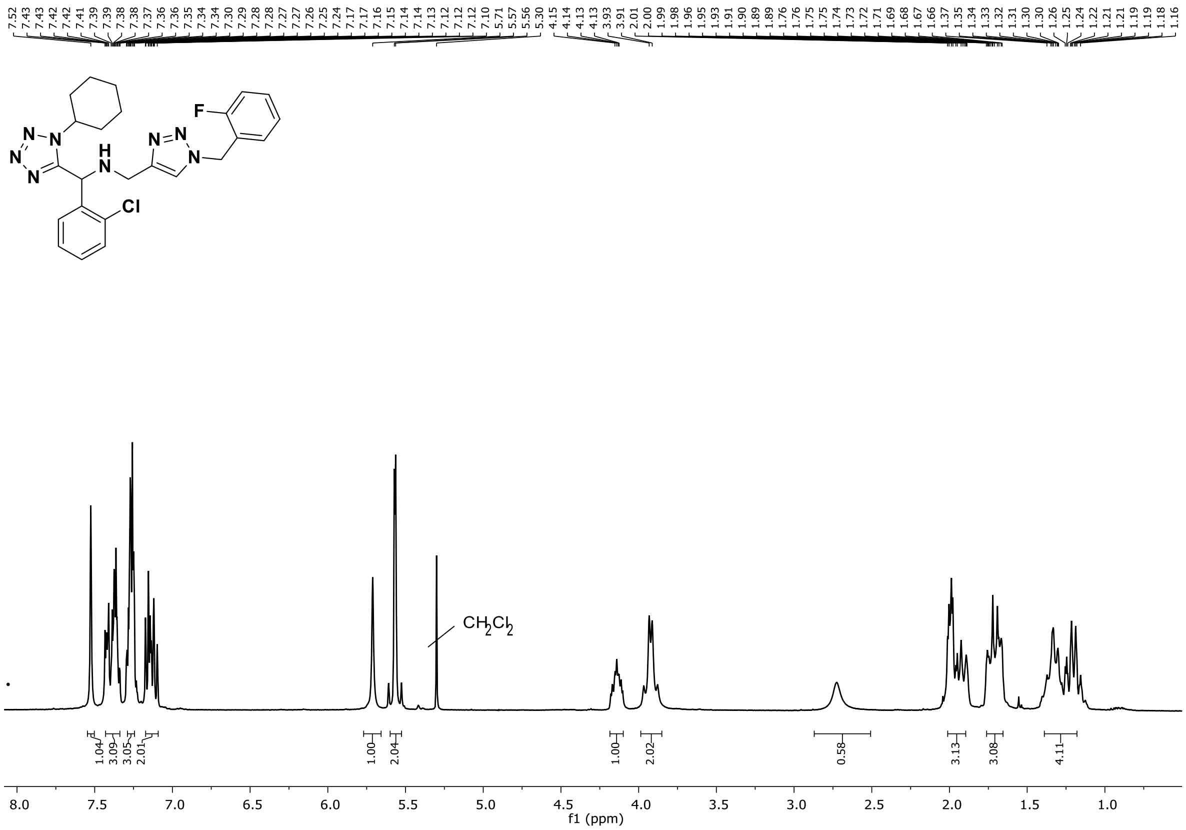

H NMR spectra of the compound **7l**.

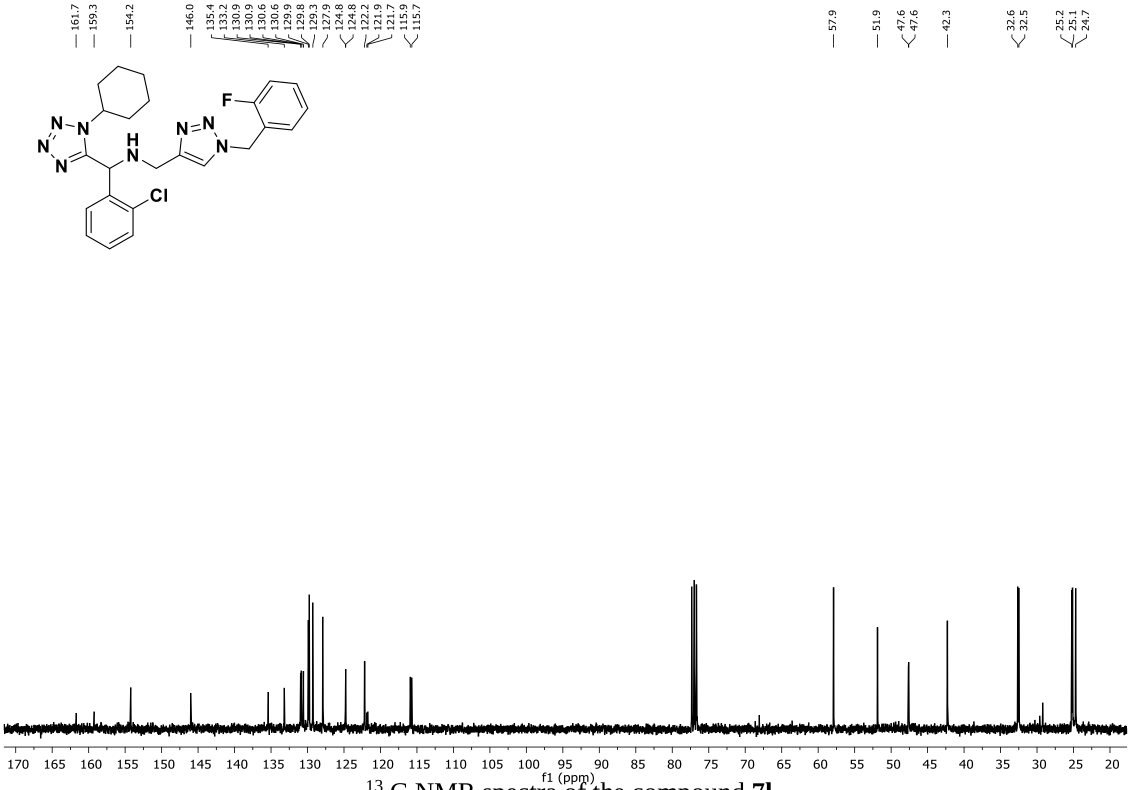

^{13}C NMR spectra of the compound **7l**.

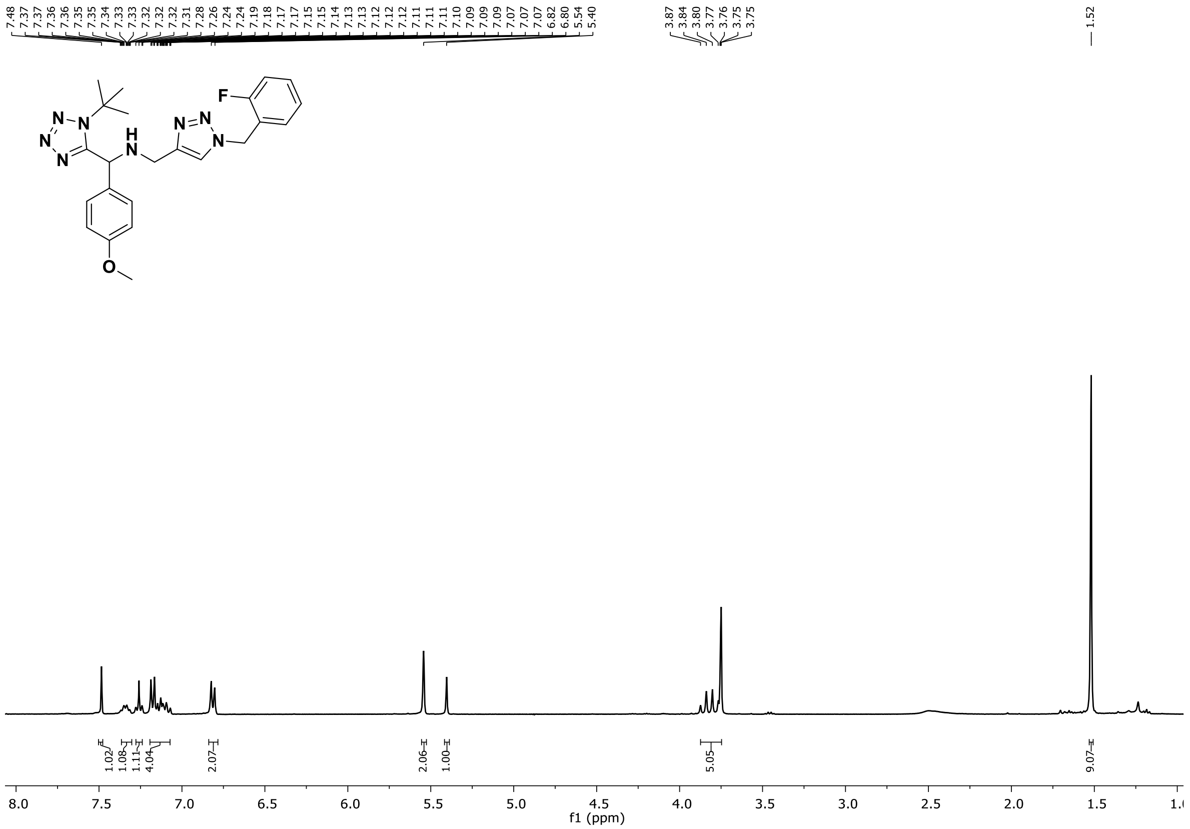

^{1}H NMR spectra of the compound **7m**.

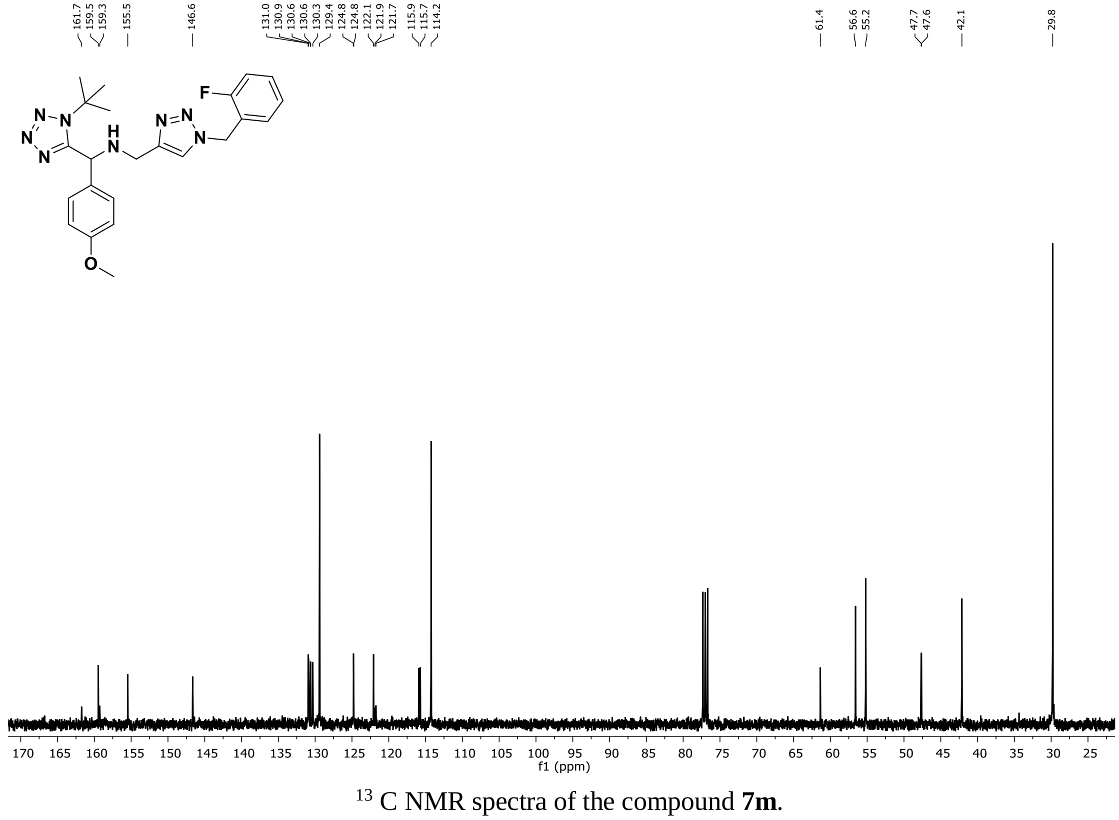

[13] C NMR spectra of the compound **7m**.

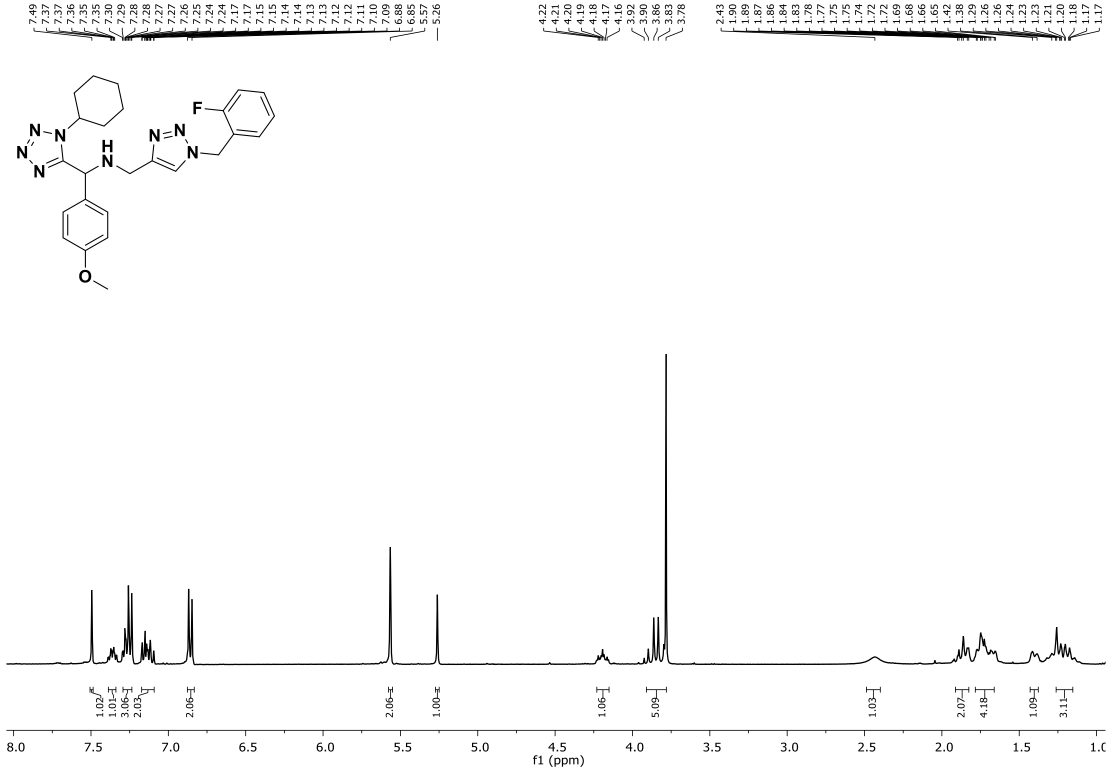

^{1}H NMR spectra of the compound **7n**.

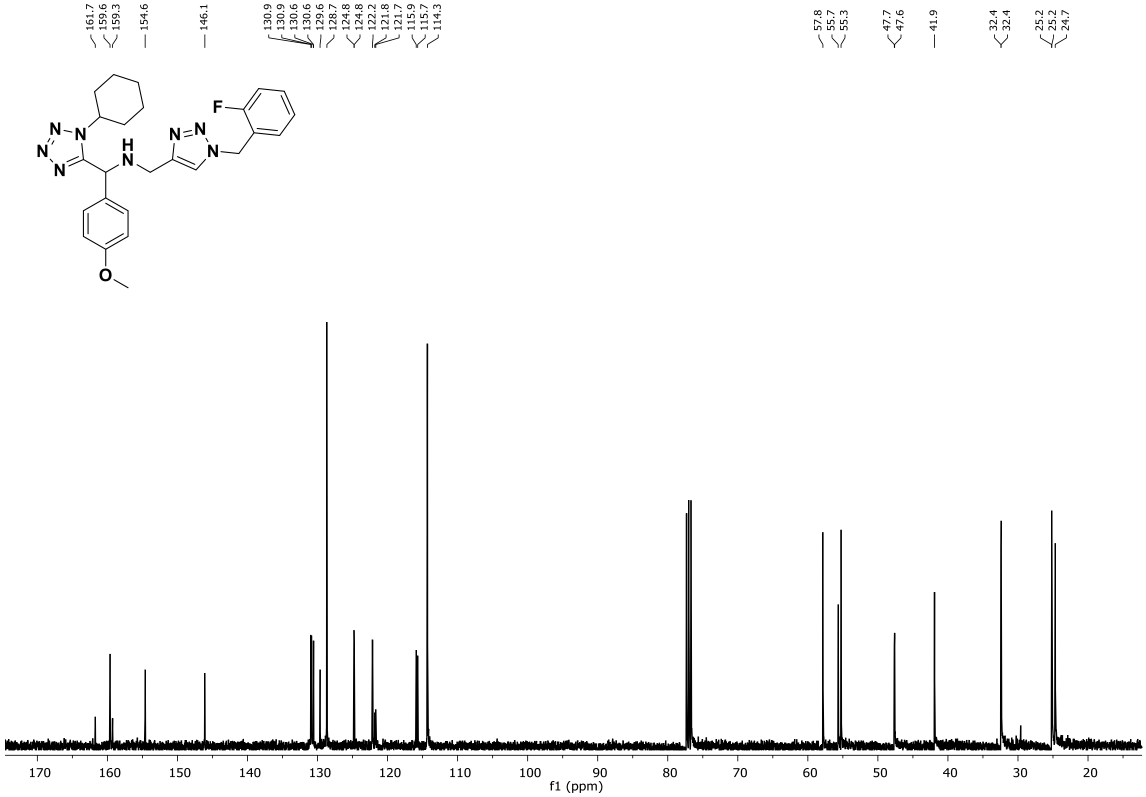

[13] C NMR spectra of the compound **7n**.

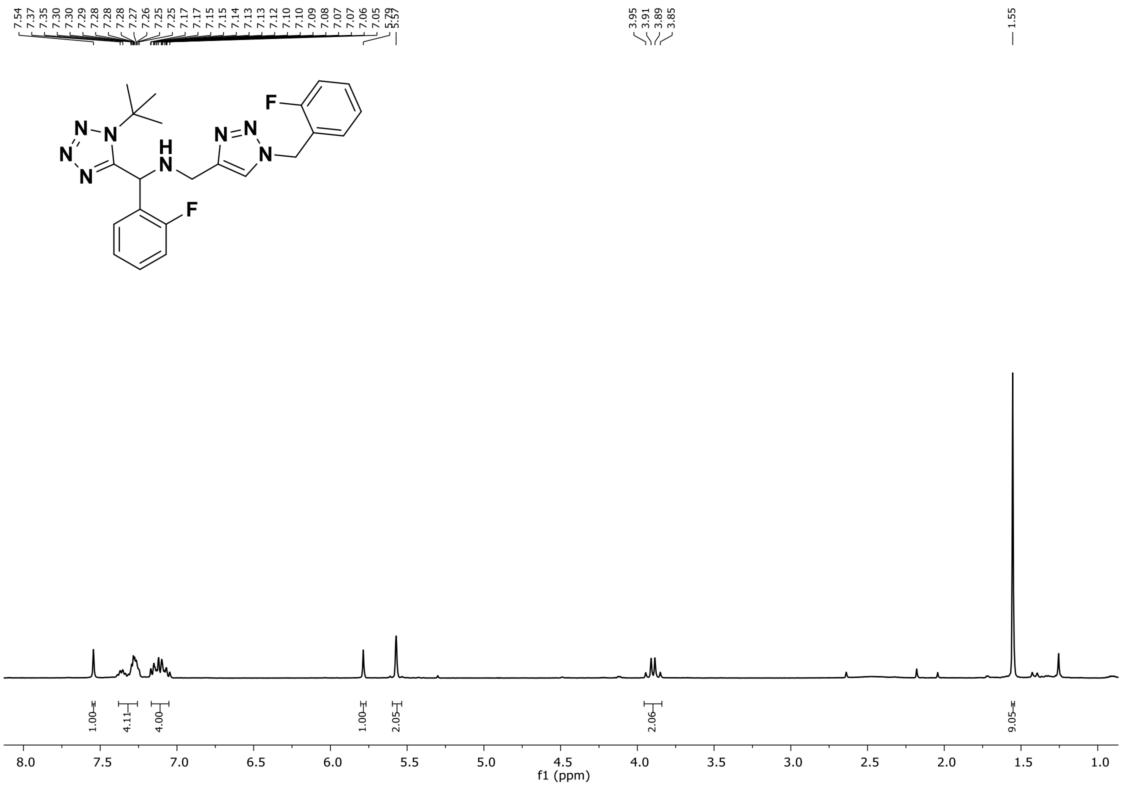

^{1}H NMR spectra of the compound **7o**.

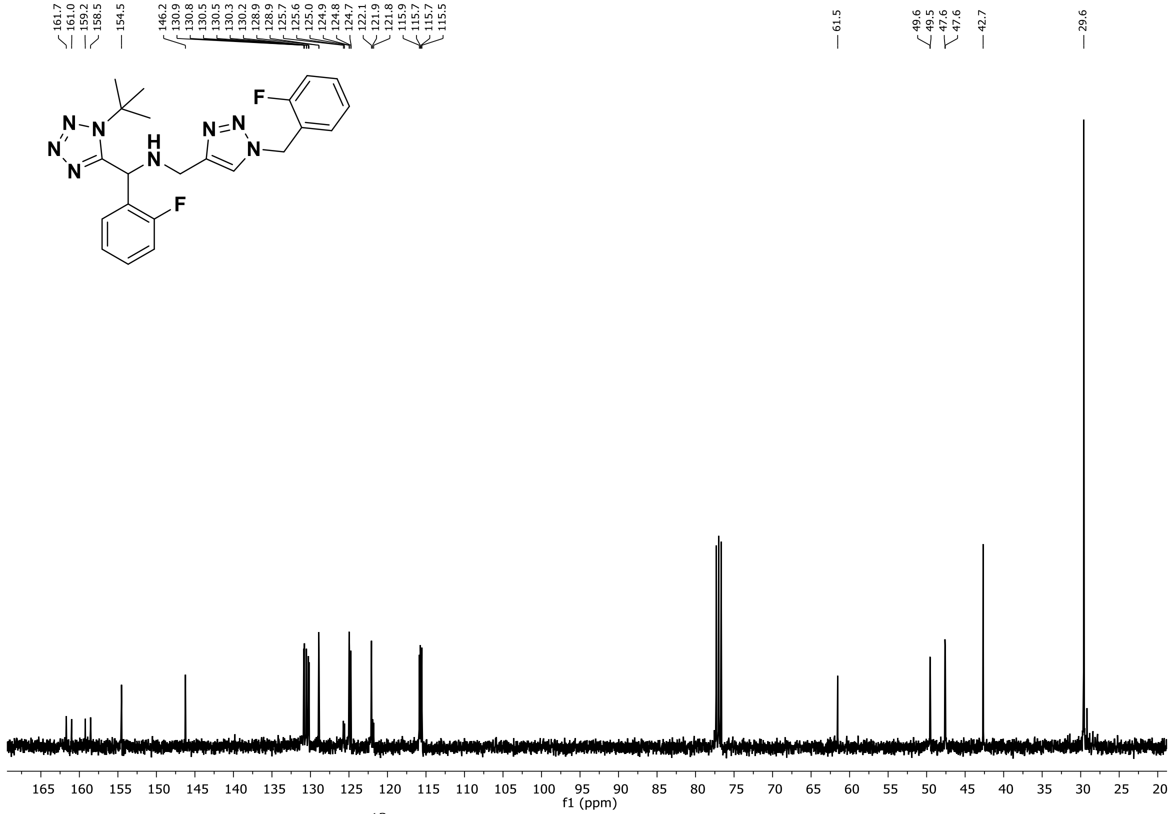

13 C NMR spectra of the compound **7o**.
